# Supplementary material for: Molecular Toxicological Mechanisms of Synthetic Cathinones on C2C12 Myoblasts
Source: Int J Mol Sci. 2019 Mar 28;20(7):1561. doi: 10.3390/ijms20071561 (PMC6479684; doi:10.3390/ijms20071561)

Supplemental file

Methods

Cell line and maintenance for cells used for western blotting

C2C12 myoblasts (American Type Culture Collection, USA) were kindly provided by Novartis (Basel, Switzerland). Cells were cultured in Dulbecco's Modified Eagle Medium – GlutaMAX supplemented with 10% fetal bovine serum (FBS) and 1% HEPES (Gibco, UK). Cells were maintained at 37°C in a humidified 5% CO_2 cell culture incubator, were passaged using trypsin upon reaching approximately 60% confluency and seeded in appropriate well plates prior differentiation into myotubes. Two days after seeding the medium was replaced by differentiation medium (DM) containing DMEM-Glutamax and 1% HEPES supplemented with 2% horse serum (Gibco, UK) and 0.029 % insulin (stock: 10mg/mL) (Sigma-Aldrich, USA) for three days. They were then starved in DMEM-Glutamax and 1% HEPES supplemented with 2% horse serum (Gibco, UK), without insulin. Myoblasts and myotubes were then treated for 24 hours with DMSO 0.1 %.

Western Blotting

C2C12 myoblasts and myotubes were lysed with RIPA buffer (150 mM sodium chloride, 1.0% NP-40, 0.5% sodium deoxycholate, 0.1% sodium dodecyl sulphate, 50 mM Tris, pH 8.0) containing complete Mini protease inhibitor cocktail (Roche Diagnostics, Mannheim, Germany). Proteins (10 µg) were resolved by SDS-PAGE using commercially available 4–12% NuPAGE Bis-Tris gels (Invitrogen, Basel, Switzerland) and transferred using the Trans-Blot Turbo Blotting System (Bio-Rad, Cressier, Switzerland). The membranes were incubated with antibodies against SOD2 (#13194, cell signaling, 1/2000), and GAPDH (sc-365062, santa cruz, 1/6000). Membranes were probed with secondary antibodies conjugated to horse radish peroxidase (HRP). Immunoblots were developed using Clarity

Western ECL Substrate (Bio-Rad Laboratories, Hercules, USA). Protein expression was quantified using the Fusion Pulse TS device (Vilber Lourmat, Oberschwaben, Germany).

Figure Legends

Suppl. Fig. 1. Oxygen consumption rate (OCR) in C2C12 cells after 1 h drug exposure. Basal respiration, leak respiration, and maximal respiration are expressed as mean ± SEM of at least three independent experiments. Drug treatments were compared to vehicle control with ANOVA followed by Dunett’s test. Significance levels are given as *p < 0.05, **p < 0.01, ***p < 0.001.

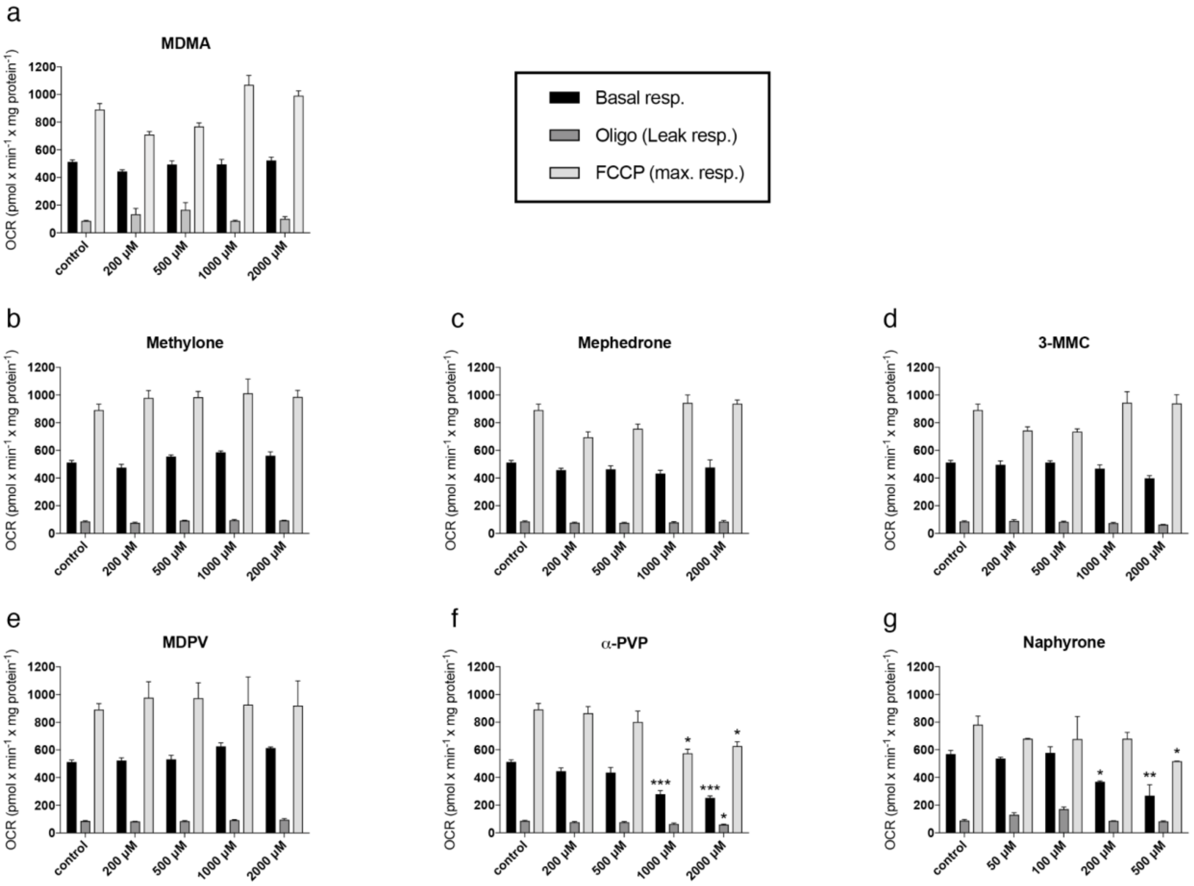

Suppl. Fig. 2. SOD2 protein expression in myoblasts and myotubes. Western blots showing the expression levels of SOD2 and GAPDH. The graph shows the quantification of SOD2 protein expression normalized against GAPDH. Data represent the mean±SEM. N= 3. * $p < 0.05$.

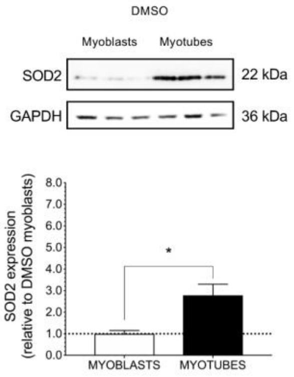

Supplement: Supplementary file 1 [file ijms-20-01561-s001.pdf]
